# Effect of Zinc Oxide Nanoparticles on Liver Functions in Albino Mice

**DOI:** 10.7759/cureus.54822

**Published:** 2024-02-24

**Authors:** Majida Jumahh Al-Ragi, Sahar S Karieb, Neila Fathallah, Amira Zaïri

**Affiliations:** 1 Department of Community Health Sciences, Ministry of Education, Baghdad, IRQ; 2 Department of Biology, College of Education for Pure Science (Ibn Al-Haitham) University of Baghdad, Baghdad, IRQ; 3 Department of Health Sciences, Faculty of Medicine (Ibn El Gazzar) University of Sousse, Sousse, TUN

**Keywords:** digestive system, kupffer cells, albino mice, liver functions, zinc oxide nanoparticles

## Abstract

Background: An alarming number of zinc oxide nanoparticles (ZnO-NPs) have leaked into the environment, endangering the tissues of many living creatures, due to the recent surge in their use in several items. Through intra-peritoneal injection, this research intends to examine the impact of ZnO-NPs on the hepatic and gastrointestinal structures of male albino mice.

Method: For seven and 14 days, animals were given 0.1 ml of 100 and 200 mg kg-1 of 50 nm-size ZnO-NPs, respectively. In contrast, those in the control group were given only water and food.

Result: The results demonstrated that the treated mice's livers underwent functional changes and histological damage. After seven and 14 days, there was a notable rise in the average levels of the glutamate-oxaloacetate transaminase and glutamate-pyruvate transaminase enzymes in comparison to the control group (p≤0.05). Concentration time determines the magnitude of this impact. When enzyme levels vary, it means the liver isn't working properly. Histological changes in the liver, such as necrosis, destruction of hepatocyte membranes, widening of sinusoidal spaces and vacuolation of their cytoplasm, vascular congestion, and an increased number of Kupffer cells, were induced in mice treated with ZnO-NPs at two studied concentrations (100 and 200 mg/kg) for seven and 14 days, respectively. These effects were time-dose-dependent, according to the results of hematoxylin-eosin staining of liver tissue images.

## Introduction

The term nanoparticles originated from the Greek term nano, meaning small or dwarf, with dimensions not exceeding 100 nanometers [1]; nanoparticles were also defined by [2] as extremely small particles having the ability to dissolve and having one or more dimensions than the external dimensions. Moreover, nanoparticles have recently received great attention due to their unique properties, which have contributed to wide areas of use such as the medical and industrial fields [3]. Furthermore, nanoparticles have been used in many industries, such as cosmetics such as sunscreens, the rubber industry, food additives, dyes, inks, paints, photographic supplies, and biosensors [4]. The third most common kind of nanoparticle is zinc oxide nanoparticles (ZnO-NPs). Their yearly output is estimated to be between 550 and 3,34,000 tons [5,6]. Therefore, these particles are likely to reach the ecosystem and affect the nature of organisms [7]. The findings of several previous studies indicated the presence of several effects of ZnO-NPs and other nanoparticles on the organs of the digestive system. Interestingly, most nanoparticles have either a harmful or a toxic effect on the liver [8], as the small size of the nanoparticles enables them to penetrate cell membranes and influence cell functions. Furthermore, their small size increases their chemical and physical properties, such as durability, chemical interaction, and conductivity [9], which enable them to affect the cells.

Because of its many functions, including bile production (which breaks down fats), storage, coagulation, and cleansing, the liver is considered a key organ in the digestive system. Any chemical compound, when exposed to it, will disrupt its operations [10]. After five days of treatment with a concentration of 10 mg/kg of ZnO-NPs, the researchers in the Slama et al. study found that the levels of aspartate aminotransferase (AST) and alanine aminotransferase (ALT) in the livers of male rats dramatically rose [11]. Consequently, the altered enzyme levels in the liver point to hepatic dysfunction accompanied by subtle morphological alterations in the liver tissues. Additionally, it was found that the accumulation of zinc nanoparticles oxidatively stressed liver tissues [12]. The findings of the study conducted by Tabish et al. also indicated that injecting the rat peritoneum with nano-porous graphene at 5 and 15 mg/kg for 27 days increased the level of liver enzymes (AST, ALP (alkaline phosphatase), and ALT), with damage to the liver and intestinal tissues [13].

Moreover, the study of Ali et al. indicated elevated AST and ALT liver enzymes due to liver dysfunction as a result of injecting rats with ZnO-NPs at a concentration of 300 mg/kg for 30 days [14]. Additionally, the study of Müller et al. revealed that the unique characteristic of these particles is their small size, which enables them to penetrate cell membranes, move from the outside to the inside, and accumulate in the liver tissues [15]. This latter, as well as their solubility, causes a range of tissue damage, leading to increased zinc ion production and concentration, cytotoxicity, oxidative stress, and liver dysfunction. Changes in the liver tissue were seen in rats administered ZnO-NPs at a dose of 5 mg/kg for 14 days. These changes included the infiltration of inflammatory cells with blood congestion, vacuolization of the hepatocyte cytoplasm, and programmed death of certain hepatocytes, as reported in the research by Mansouri et al. [16]. In a similar vein, rats given 2 mg/kg of copper nanoparticles for three days had vascular injury and necrosis as a result of a reduction in the quantity of aqueous fluid around the liver portal veins [17]. Consequently, the purpose of this research is to investigate how ZnO-NPs affect the functional alterations and histological structure of albino mouse livers.

## Materials and methods

Animal collection and studied group characteristics

**Figure 1:**
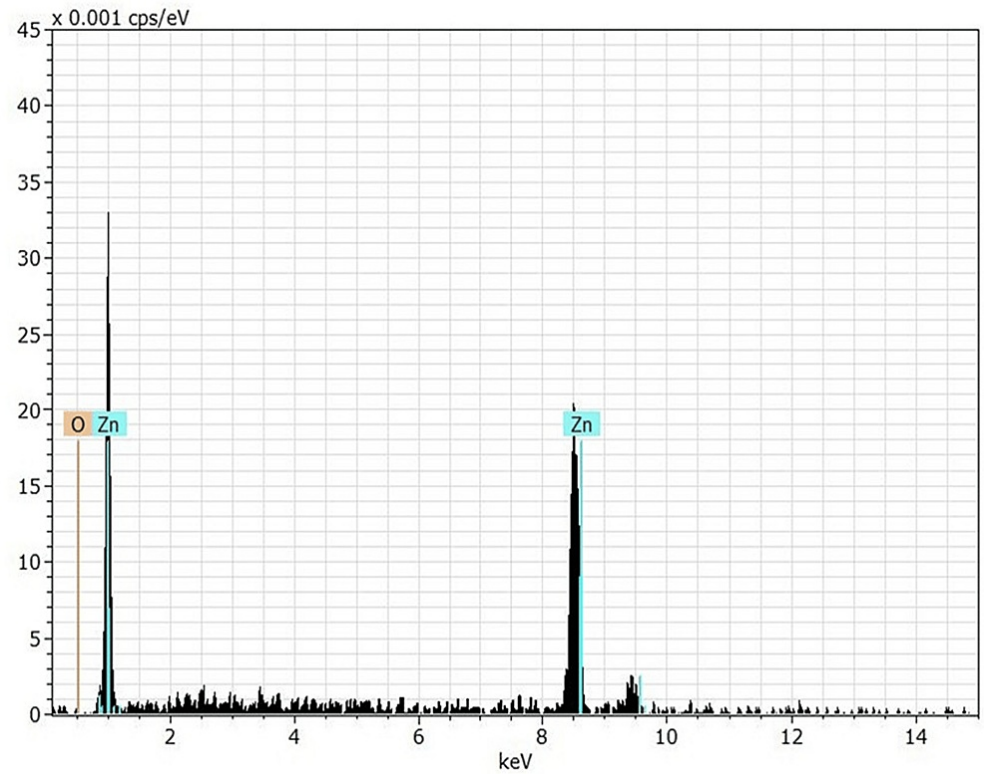
Purity of ZnO-NPs using the SDX device ZnO-NPs: zinc oxide nanoparticles, SDX: energy dispersive X-ray spectroscopy

The research used 40 Balb/C strain Swiss white mice that were procured from the Industrial Research and Development Authority. The ethical number for the study was CEPS/IEC/2022/1 from the College of Education for Pure Science. With an average weight of 30-35 grams and an age of 8-12 weeks, these were healthy and happy. A private laboratory's animal home was the next stop, where the animals were housed in plastic cages covered with wire mesh. Cages were cleaned and sanitized daily during the trial, and sawdust was added to them every two days. The animals were provided with water and food and kept in a controlled environment with 12 hours of darkness and 12 hours of light, as well as a temperature range of 20-30°C and enough ventilation. Zhengzhou Dongyao Nano Materials Co., Ltd.'s ZnO-NPs were used; these particles exhibited the following characteristics: a white powder appearance, an average size of 50 nanometers, a cubic shape, and a particle purity level of 99.99%. Also, as shown in Figure 1, the powder's purity was certified to be 100% utilizing the energy dispersive X-ray spectroscopy (SDX) equipment at Al-Nahrain University, College of Science, Department of Physics, Electron Microscope Laboratory.

**Figure 2:**
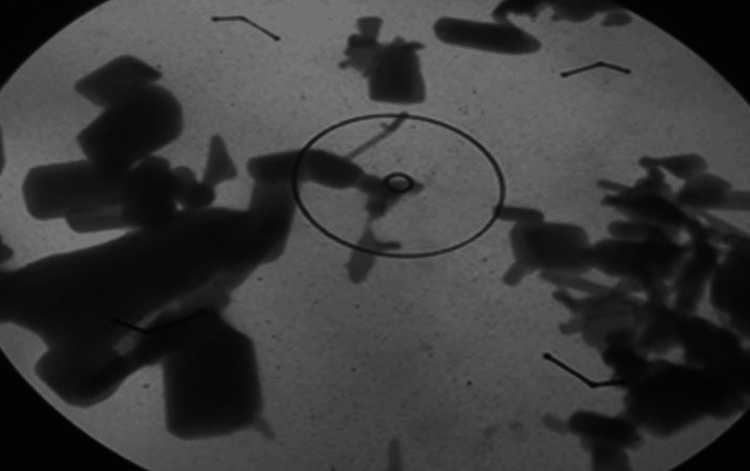
Cubic shapes of ZnO-NPs using TEM microscope ZnO-NPs: zinc oxide nanoparticles, TEM: transmission electron microscopy

The transmission electron microscopy facility at Al-Nahrain University, College of Medicine, was used to detect the size and shape of the nanoparticles under study. The particles were found to be cubical in shape, with some forming clusters (Figure 2), which was in close agreement with the specifications provided by the manufacturer.

Two concentrations of 100 and 200 mg/kg of ZnO-NPs powder were generated for the dosing solution by dissolving 325 and 650 mg of this powder in 13 ml of distilled water, respectively. After that, a storage bottle with a vial was used to combine the ingredients for each concentration. The tube was set on a magnetic stirrer for 30 minutes to ensure thorough mixing. To avoid any possible agglomeration, the mixture was then subjected to an ultrasonic sterilizer for 15 minutes. The ZnO-NPs solutions are mixed daily, and then the animals are given a dose by putting the mixture on a magnetic stirrer for 15 minutes. The animals are then orally administered, using a plastic tube, 0.1 ml of the produced solution from each concentration of ZnO-NPs once daily for the first seven days and again for the second 14 days of treatment in the following dosages.

Eight male white mice were given 0.1 milliliters of distilled water in the first set of experiments. This set of people served as the reference group. In the second set of tests, eight male white mice were given 0.1 milliliters of ZnO-NPs at a concentration of 100 mg/kg for seven days. Eight male white mice participated in the third group; they were given 0.1 ml of ZnO-NPs at a concentration of 100 mg/kg for 14 days. Eight male white mice constituting the fourth group were given 0.1 ml of ZnO-NPs at a concentration of 200 mg/kg for seven days. As for the fifth set of subjects, eight male white mice were given 0.1 ml of ZnO-NPs at a concentration of 200 mg/kg and left to recover for 14 days.

Biochemical analysis and histological section preparation

After 14 days of the experiment, the animals were given chloroform to make them unconscious, and blood samples were taken by puncturing their hearts. Hepatic enzyme activity, including ALT, AST, glutamate-oxaloacetate transaminase (GOT), and glutamate-pyruvate transaminase (GPT), was assessed by centrifugation of the serum separated at 5000 rpm for five minutes after samples were collected into tubes containing a gelatinous substance (gel tube). After that, the animals were rendered limp using the cervical dislocation technique, and the liver was promptly removed and rinsed with normal saline (0.9%). Using a spectrophotometer (Milton Roy Spectronic 1201), the activity of plasma ALT and AST was measured using colorimetric enzymatic techniques (Biodiagnostic kits).

After the liver was excised, it was fixed in a 10% formalin solution for 48 hours. The fixative was then rinsed out of the liver using tap water many times. Finally, it was preserved in 70% ethyl alcohol for histological section preparation. A light microscope with magnifications of 10 and 40X was used to view all of the liver histology slides. The next step was to capture pictures of various liver histology sections using a laptop and an Omax USB Camera (3.7, China) attached to an Olympus compound microscope.

Statistical analysis

We used SPSS Statistics version 24 (IBM Corp. Released 2016. IBM SPSS Statistics for Windows, Version 24.0. Armonk, NY: IBM Corp.), a statistical application for the social sciences, to conduct a one-way ANOVA test on all of the data. We then calculated the LSD, or least significant difference, between the means of the groups that were examined. Data were shown as mean±SE (standard error), and differences were judged significant at p≤0.05.

## Results

The present research found that, compared to the control group, animals treated with ZnO-NPs had histological alterations and an increase in the activity level of liver enzymes.

Biochemical changes

Activity Levels of Glutamate-Oxaloacetate Transaminase (GOT/AST)

The statistical analysis showed that there was a significantly different amount of GOT (p<0.0001) for both the seven-day and 14-day groups, as well as for the two concentrations (100 and 200 mg/kg; Table 1). Curiously, mice given ZnO-NPs at a dosage of 100 mg/kg for 14 days had a much different GOT level (126.75±1.71 mg/dL) compared to animals given 200 mg/kg for seven days (116.79±24.48 mg/dL). On average, a concentration of 267.46±14.25 mg/dL was observed in animals given a 200 mg/kg dose for 14 days, compared to 25.30±2.44 mg/dL in the control group. The only exception to this was the group of animals given a 100 mg/kg dose for seven days, whose average concentration was 45.98±4.23 mg/dL.

As a bonus, there were notable distinctions between the two groups of rats given doses of 200 and 100 mg/kg for seven days and between the two groups given doses of 200 and 100 mg/kg for 14 days.

In addition, there was a notable difference between the two groups of animals given 100 mg/kg for seven and 14 days, as well as between the groups given 200 mg/kg for two and 14 days. Table 1 shows that after seven days of treatment at a dose of 100 mg/kg, the GOT enzyme levels varied significantly across all tested groups of animals.

**Table 1:** Activity level of GOT enzyme after treatment with 100 and 200 mg/kg of ZnO-NPs for two periods (seven and 14 days) * significant difference ZnO-NPs: zinc oxide nanoparticles, GOT: glutamate-oxaloacetate transaminase, SE: standard error

Groups treated with ZnO-NPs	GOT level (mg/dL) (mean±SE)	p-value
Control	25.30±2.44	<0.0001*
ZnO-NPs (100 mg/Kg) 7 days	45.98±4.23	
ZnO-NPs (100 mg/ Kg) 14 days	126.75±1.74	
ZnO-NPs (200 mg/ Kg) 7 days	116.79±24.48	
ZnO-NPs (200 mg/ Kg) 14 days	267.46±14.25	

Activity Levels of Glutamate-Pyruvic Transaminase (GPT/ALT)

The results of the statistical analysis indicated significant differences (p=0.05) in the level of GPT enzyme among all groups of animals treated with ZnO-NPs for the two studied concentrations (100 and 200 mg/kg) and for the periods of seven and 14 days (Table 2). Moreover, a high significant difference appeared in the level of GPT for animals treated with a concentration of 200 mg/kg after 14 days, as the mean was 46.03±6.84 in comparison to the mean of the control group, which was 29.99±2.56 mg/dL. We also found significant differences with animals treated at a concentration of 100 mg/kg for 14 days, as the mean was 32.81±1.97 mg/dL, as well as significant differences with a group of animals treated at a concentration of 100 mg/kg for seven days, for which the mean was 31.04±1.30 mg/dL. It also had significant differences with the group of animals treated with a concentration of 200 mg/kg for seven days (the mean was 35.86±4.58 mg/dL), as shown in Table 2.

**Table 2:** Activity level of GPT enzyme after treatment with 100 and 200 mg/kg of ZnO-NPs for two periods (seven and 14 days) * Significant differences between groups in general vs. control group ZnO-NPs: zinc oxide nanoparticles, GPT: glutamate-pyruvate transaminase, SE: standard error

Groups treated with ZnO-NPs	GPT level (mg/dL) (mean±SE)	p-value
Control	29.99±2.56	0.05*
ZnO-NPs (100 mg/Kg) 7 days	31.04±1.30	
ZnO-NPs (100 mg/ Kg) 14 days	32.81±1.97	
ZnO-NPs (200 mg/ Kg) 7 days	35.86±4.58	
ZnO-NPs (200 mg/ Kg) 14 days	46.03±6.84	

Histological changes in the liver

**Figure 3:**
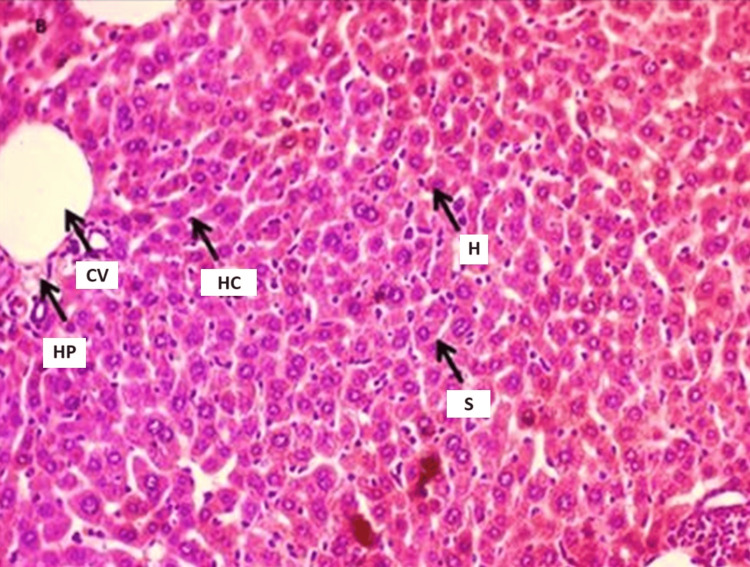
Cross-section of the liver of animals treated with a concentration of 100 mg/kg of ZnO-NPs for seven days H: hepatocytes, HP: hepatic pylorus, GH: Glisson’s capsule, CV: central vein, PV: hepatic portal vein, HC: hepatic chords, S: hepatic sinuses

The microscopic examinations of the mice's liver in the control group using hematoxylin-eosin stain showed the normal structure of the liver, as the hepatocytes appeared in a regular and compact hexagonal shape, interspersed with hepatic sinusoids containing blood vessels, a section of the pyloric area, and Glisson’s capsule. The cross sections of mouse liver tissue treated with 100 mg/kg of ZnO-NPs for seven days showed a set of histological changes, including necrosis, some degenerated hepatocytes with destruction of their membranes, widened sinusoidal spaces, and congested blood vessels (Figure 3).

Furthermore, mice exposed to the same substance for 14 days showed increased hepatocyte necrosis and membrane destruction, increased sinusoidal space widening, and vascular congestion as compared to the normal tissue of the liver of mice. Additionally, the cross sections in the mice liver treated with a concentration of 200 mg/kg of ZnO-NPs for seven days noticed clustering and infiltration of mononuclear inflammatory cells around blood vessels. When increasing the exposure time to 14 days, the treated animals with the same concentration showed an increased number of Kupffer cells in the space of sinusoids, as well as an increased infiltration of inflammatory cells. On the other hand, the blood vessels appeared more dilated, with hemorrhage inside the vessel. In addition to the associated destruction of the blood vessel walls, the histological changes were obvious compared to the seven-day exposure period at the same concentration of ZnO-NPs.

## Discussion

Enzymatic functions in the liver

In this investigation, GOT and GPT enzyme levels were shown to be raised in the blood of mice that were treated with ZnO-NPs at dosages of 100 and 200 mg/kg for seven and 14 days, respectively. It appears that the longer and more concentrated this impact is, the more noticeable it becomes. A high concentration of these enzymes is indicative of liver disease [18,19]. The inflammation caused by hepatocyte degeneration and necrosis increases the amount of permeability of their membranes, which may explain why there are high levels of liver enzymes (GOT and GPT). Consistent with previous research, our study found that GPT and GOT enzyme levels in mice were increased after seven and nine days of graphene nanoparticle administration at a dose of 60 mg/kg [20]. In addition, this study's results were in line with those of Tabish et al., who found that rats exposed to nano-porous graphene injections into their peritoneum for 27 days at concentrations of 5 and 15 mg/kg had elevated levels of liver enzymes (AST, ALT, and ALP) and histological damage to their livers and intestines [13]. The research found that the increased enzymes are caused by liver tissue damage.

Additionally, the study by Ali et al. indicated that the level of liver hepatic enzymes, including AST and ALT, increased due to liver dysfunction in the liver secondary to injecting rats with ZnO-NPs at a concentration of 300 mg/kg for 30 days [14]. In contrast, the study of Doudi and Setorki observed that the toxic effects of nanoparticles stimulate active oxygen reactions, which produce free radicals that destroy liver cells, inducing elevated secretion of hepatic enzymes into the circulatory system [21]. After liver tissue damage, this negatively affects the hepatocytes that lose the permeability properties of their plasma membrane, leading to more severe infiltration of more liver enzymes into the circulatory system and an increase in their blood levels in the blood [22].

The results of this study showed, through the cross sections of mice liver treated with ZnO-NPs at increasing concentrations (100 and 200 mg/kg) and durations (seven and 14 days) using hematoxylin-eosin stain, the occurrence of histological abnormalities represented by necrosis, degeneration, and destruction of some liver cells and their membranes, a widening of sinus spaces with congestion of blood vessels, and clumping and infiltration with inflammatory cells. These changes seem to increase with increasing concentration and duration. The reason for this may be explained by the properties of nanoparticles, which are smaller than 100 nanometers, facilitating their penetration into the animal’s organs, and this was observed as reported in the study of Gilmour et al. [23]. Additionally, ZnO-NPs also have a high cellular solubility and accumulation when they enter cells and accumulate, increasing positive zinc ion production and their concentration, causing cytotoxicity and oxidative stress [15]. The observations of the current study agreed with the study of Mansouri et al. revealing that the treatment of rats with ZnO-NPs at a concentration of 5 mg/kg for 14 days changed the liver tissue, represented by infiltration of inflammatory cells with blood congestion, vacuolization of the hepatocyte cytoplasm, and programmed death of some hepatocytes [16].

Contrary to what one would expect from a control group, this research found that rats given ZnO-NPs for seven or 14 days had wider and more congested blood channels in their liver tissues. This discovery might be a result of hypertension and the harmful effects of ZnO-NPs on the epithelial layer that lines blood vessels, which in turn causes inflammation in the liver. These findings corroborated those of previous research by Hosseini et al. [24] that examined the effects of injecting ZnO-NPs into the peritoneum of adult female mice over the course of 30 days at progressively higher doses (4, 8, 25, 50, 100, and 200 mg/kg). Liver necrosis, inflammatory cell infiltration, and blood vessel congestion were the results. The effects were more pronounced when the concentrations were raised. In addition, histological sections of livers from mice treated with 100 and 200 mg/kg of ZnO-NPs for seven and 14 days revealed hepatocytes that were divided or binucleated. Because the liver reacts to nanoparticle toxicity by trying to replenish damaged or dead hepatocytes via cell division, this is the case. This finding is in line with the research of Gerlyng et al., which indicated that an increase in divided or binucleated hepatocytes is the liver's way of trying to repair new cells rather than damaged ones [25].

The study has some limitations; while it highlights potential harm caused by ZnO-NPs, the use of animal models, such as mice, may not perfectly mimic human responses, warranting caution in extrapolating these results to human health. Furthermore, the study's focus on short-term exposure through intra-peritoneal injection leaves unanswered questions about the long-term or chronic effects of ZnO-NPs and their cumulative impact.

## Conclusions

The digestive organs of adult white mice were shown to be adversely impacted by ZnO-NP treatment for seven and 14 days at doses of 100 and 200 mg/kg, according to the results of the present investigation. Because these effects were seen on both the morphological appearance of the liver cells and the alterations in the function and histological structure of the liver, they intensified with increasing concentrations of ZnO-NPs and with extended oral administration. Given the wide range of applications for ZnO-NPs in both animals and people, it was proposed that future research should employ lower concentrations of the particles to establish their safe upper limit.

## References

[1] Kumah EA, Fopa RD, Harati S, Boadu P, Zohoori FV, Pak T (2023). Human and environmental impacts of nanoparticles: a scoping review of the current literature. BMC Public Health.

[2] Nikalje AP (2015). Nanotechnology and its application in medicine. Med Chem.

[3] Tyner KM, Roberson MS, Berghorn KA, Li L, Gilmour RF Jr, Batt CA, Giannelis EP (2004). Intercalation, delivery, and expression of the gene encoding green fluorescence protein utilizing nanobiohybrids. J Control Release.

[4] Guerra FD, Attia MF, Whitehead DC, Alexis F (2018). Nanotechnology for environmental remediation: materials and applications. Molecules.

[5] Connolly M, Fernández M, Conde E, Torrent F, Navas JM, Fernández-Cruz ML (2016). Tissue distribution of zinc and subtle oxidative stress effects after dietary administration of ZnO nanoparticles to rainbow trout. Sci Total Environ.

[6] Peng YH, Tsai YC, Hsiung CE, Lin YH, Shih YH (2017). Influence of water chemistry on the environmental behaviors of commercial ZnO nanoparticles in various water and wastewater samples. J Hazard Mater.

[7] Rajput VD, Minkina TM, Behalb A (2018). Effects of zinc-oxide nanoparticles on soil, plants, animals and soil organisms: a review. Environ Nanotechnol Monit Manag.

[8] Sharma V, Singh P, Pandey AK, Dhawan A (2012). Induction of oxidative stress, DNA damage and apoptosis in mouse liver after sub-acute oral exposure to zinc oxide nanoparticles. Mutat Res.

[9] Nel A, Xia T, Mädler L, Li N (2006). Toxic potential of materials at the nanolevel. Science.

[10] Ponder KP, Gupta S, Leland F (1991). Mouse hepatocytes migrate to liver parenchyma and function indefinitely after intrasplenic transplantation. Proc Natl Acad Sci U S A.

[11] Slama IB, Amara S, Mrad I (2015). Sub-acute oral toxicity of zinc oxide nanoparticles in male rats. J Nanomed Nanotech.

[12] Fujihara J, Tongu M, Hashimoto H, Fujita Y, Nishimoto N, Yasuda T, Takeshita H (2015). Pro-inflammatory responses and oxidative stress induced by ZnO nanoparticles in vivo following intravenous injection. Eur Rev Med Pharmacol Sci.

[13] Tabish TA, Pranjol MI, Jabeen F (2018). Investigation into the toxic effects of graphene nanopores on lung cancer cells and biological tissues. Appl Mater Today.

[14] Ali RM, Abbas NK, Abbas AK, Abbas LK (2020). Histological sections of pancreas and serum biochemical changes in rats after dexamethasone and zinc oxide nanoparticles injection. Med Legal Update.

[15] Müller KH, Kulkarni J, Motskin M (2010). pH-dependent toxicity of high aspect ratio ZnO nanowires in macrophages due to intracellular dissolution. ACS Nano.

[16] Mansouri E, Khorsandi L, Orazizadeh M, Jozi Z (2015). Dose-dependent hepatotoxicity effects of zinc oxide nanoparticles. Nanomed J.

[17] Sizova EA, Miroshnikov SA, Poliakova VS, Lebedev SV, Glushchenko NN (2013). Copper nanoparticles as modulators of apoptosis and structural changes in some organs (Article in Russian). Morfologiia.

[18] Patlolla AK, Berry A, Tchounwou PB (2011). Study of hepatotoxicity and oxidative stress in male Swiss-Webster mice exposed to functionalized multi-walled carbon nanotubes. Mol Cell Biochem.

[19] Patlolla A, McGinnis B, Tchounwou P (2011). Biochemical and histopathological evaluation of functionalized single-walled carbon nanotubes in Swiss-Webster mice. J Appl Toxicol.

[20] Singh T, Sinha N, Singh A (2013). Biochemical and histopathological effects on liver due to acute oral toxicity of aqueous leaf extract of Ecliptaalba on female Swiss albino mice. Indian J Pharmacol.

[21] Doudi M, Setorki M (2014). Acute effect of nano-copper on liver tissue and function in rat. Nanomed J.

[22] Aneja S, Vats M, Aggarwal S, Sardana S (2013). Phytochemistry and hepatoprotective activity of aqueous extract of Amaranthus tricolor Linn. roots. J Ayurveda Integr Med.

[23] Gilmour PS, Ziesenis A, Morrison ER (2004). Pulmonary and systemic effects of short-term inhalation exposure to ultrafine carbon black particles. Toxicol Appl Pharmacol.

[24] Hosseini SM, Amani R, Moshrefi AH, Razavimehr SV, Aghajanikhah MH, Sokouti Z (2020). Chronic zinc oxide nanoparticles exposure produces hepatic and pancreatic impairment in female rats. Iran J Toxicol.

[25] Gerlyng P, Abyholm A, Grotmol T, Erikstein B, Huitfeldt HS, Stokke T, Seglen PO (1993). Binucleation and polyploidization patterns in developmental and regenerative rat liver growth. Cell Prolif.

